# The Treatment of Pterygia with the Galvano-Cautery

**Published:** 1894-07

**Authors:** Arthur G. Hobbs

**Affiliations:** Atlanta, Ga.


### THE TREATMENT OF PTERYGIA WITH THE GALVANO-CAUTERY.*

By Arthur G. Hobbs, M.D.,
Atlanta, Ga.

I shall not presume that it is necessary in describing this method of operating on pterygia to even enumerate the many already in vogue. I should rather ask your forbearance for merely suggesting to you that yet another method is to be added to the list already, perhaps, too long; still it may be true that each of these operations has served its purpose in turn in the process of evolution. Since it is the cardinal point in making a pterygium operation to prevent two raw surfaces from remaining in apposition after the operation, which necessarily results in the relapses which we too frequently see, why not then convert each of the raw surfaces into an eschar at once to avoid such failures, provided we are able to demonstrate practically that this means will bring about that desired result?

For some years the galvano-cautery point has been resorted to in corneal ulcers, particularly in those indolent and intractable ulcers which have refused to yield to other methods of treatment. It was suggested to me a few years ago, after seeing some of the beautiful results of the cautery when applied to ulcers of the cornea, that it could, at least, produce no bad results if properly applied to the

*Read before the Georgia Medical Association, April, 1894.

neck of a pterygium for the purpose of cutting off the nutrition of its corneal apex. I hoped that it would prove a better means of severing the arterial supply to the apex as it crossed the sclero-corneal junction. This object is generally attained by the use of the knife, and various methods have been resorted to to accomplish the purpose. The failures in pterygium operations are usually due to the resulting re-establishment of the arterial circulation, and when this occurs the desired end of the operation fails in proportion to the number of the re-established vessels. The great vascularity of a pterygium, with its vessels crossing over the sclero-corneal line to supply its apex on the cornea, renders it difficult to permanently sever all the arteries by a clean cut with the knife; hence, perhaps, a score of different methods of knife and scissors operations have been resorted to by different operators to accomplish this end.

Each operator aims at one end primarily, and that is to completely cut off all the blood supply from the corneal apex. Secondarily, his desire is to destroy as little conjunctiva as possible. When the first end is attained the corneal part of the pterygium has lost its direct nutrition and can then only share with the cornea proper for its sustenance, which is indirect, or by imbibition. As this meager supply proves insufficient atrophy results, and the partial, or almost complete, obliteration after a time will depend upon the activity of the absorbents. For this reason the best results are obtained in younger subjects in which, as a rule, the lymphatics are more active, as is also the contractility of the arterial coats.

In many cases when the knife is resorted to a secondary pterygium results, in which sufficient nutrition is re-established to the apex to perpetuate the corneal haziness even beyond its apparent margin to greatly interfere with vision. When the cautery section is well made I have not seen these secondary results that are often so difficult to prevent by a clean cut with the knife or scissors. In some cases, however, it may be best to combine the methods of operating, as I have done in two cases not included in this report. These two pterygia were extremely vascular and muscular; hence I first used the knife in the usual way, and then seared the divided edges with the cautery blade to prevent the secondary results.

The fine pointed cautery blade, heated with a battery that can be perfectly gauged and always relied on, is applied horizontally to the narrowest portion of the growth, which is near the sclero-corneal line; the touch is made at the moment white heat is reached and should be almost instantaneous and reapplied as quickly if the tissues are not at first completely severed. If the growth is not adherent to the sclera and corneal margin, it is best to grasp it with small forceps and slightly raise it from the sclera. When the forceps are used it is easier to be certain that the cautery has made a complete section of all the hypertrophied and over-vascular conjunctival tissue.

Where the corneal head is large and protruding it is advisable to make a cautery application in the same manner as in applying it to a corneal ulcer. In both cases the resulting cicatrix is less—it is more transparent. This is not altogether an unknown fact, but one that has seemed never to have been brought out as prominently as its importance would suggest. For this reason one may be tempted often to resort to the cautery to reduce a large pterygium apex or a corneal ulcer, for the purpose alone of attaining this corneal transparency. The resulting eschar so completely severs the vascular connection, leaving no raw surfaces in apposition, that a re-established circulation is practically impossible; this cannot be said when the knife or scissors have been used. In case the redundancy of tissue at the point of section should seem to require reduction, the scleral end of the section may be turned under and transvere suture introduced.

I have made this operation fourteen times, and would have made it half as many more times during the last two years but for that decided objection to the cautery which we so often meet with and are bound to respect. This operation is more quickly performed; it is followed by no bleeding; it leaves no ecchymosis; the wound heals more rapidly; there is less pain during and after its performance; it requires no bandages and the corneal haziness around the apex is less.

A weak solution of cocaine, about a two per cent., is first dropped into the conjunctival sac, then after about five minutes a stronger solution, about a ten per cent., is applied at intervals, locally, by means of a small cotton probe, for five minutes more; in the meantime the lids are held open. By this means the toxic effects are avoided except in those subjects that are unusually susceptible to cocaine. In using cocaine in this latter strength, I try to avoid as much as possible, by the position of the patient, its contact with the cornea. It is now a well known fact that when used in this strength, or even when much weaker, that this drug is disastrous to an abraded cornea, if long continued.

The kind of galvano-cautery apparatus, the ease of its manipulation, the size of its blade, the confidence of the hand that presses the button, the requisite degree of heat, which should be gauged by the turn of the screw, are some of the necessary factors in this seemingly simple, yet delicate, little operation, whether it be made to a corneal ulcer or to the neck of a pterygium. The only galvano-cautery apparatus in my knowledge that I would now be willing to trust for these purposes is the "Transformer" of the alternating current of fifty-two volts.

I am now using, in my consultation rooms, the first two of these little instruments made. I described the various uses and advantages of this "Transformer" in a paper read before the Pan-American Congress last fall. So far as I know, the galvano-cautery has not hitherto been used as a means of cutting off the corneal apex of a pterygium.

